# The pharmacokinetics of buserelin after intramuscular administration in pigs and cows

**DOI:** 10.1186/s12917-022-03237-0

**Published:** 2022-04-11

**Authors:** Jingyuan Kong, Fuqin Su, Yu Liu, Yuxin Yang, Yuying Cao, Jicheng Qiu, Yue Wang, Lu Zhang, Jianzhong Wang, Xingyuan Cao

**Affiliations:** 1 Department of Veterinary Pharmacology and Toxicology, College of Veterinary Medicine, China Agricultural University, Yuanmingyuan West Road 2#, Beijing, 100193 China; 2 China Institute of Veterinary Drug Control, Beijing, China; 3 Shanxi key lab. for modernization of TCVM, College of Veterinary Medicine, Shanxi Agricultural University, Mingxian South Road 1#, Taigu, Shanxi, 030801 China; 4 Key Laboratory of Detection for Veterinary Drug Residues and Illegal Additives, Ministry of Agriculture and Rural Affairs of the People’s Republic of China, Beijing, China

**Keywords:** Pharmacokinetics, Buserelin, Pigs, Cows, Intramuscular, UPLC-MS/MS

## Abstract

**Background:**

Buserelin is a luteinizing hormone releasing hormone (LHRH) agonist used for the treatment of hormone-dependent diseases in males and females. However, the pharmacokinetics of buserelin in pigs and cows are not fully understood. This study was designed to develop a sensitive method to determine the concentration of buserelin in blood plasma and to investigate the pharmacokinetic parameters after intramuscular (i.m.) administration in pigs and cows.

**Results:**

A sensitive and rapid stability method based on ultra-performance liquid chromatography tandem mass spectrometry (UPLC-MS/MS) was developed. The pharmacokinetic parameters of buserelin after i.m. administration were studied in five pigs and five cows at a single dose of 1 mg per pig and 3 mg per cow. The plasma kinetics were analyzed by WinNonlin 8.1.0 software using a non-compartmental model. The mean concentration area under the curve (AUC_0-t_) was 25.02 ± 6.93 h × ng/mL for pigs and 5.63 ± 1.86 h × ng/mL for cows. The maximum plasma concentration (C_max_) and time to reach the maximum concentration (t_max_) were 10.99 ± 2.04 ng/mL and 0.57 ± 0.18 h for pigs and 2.68 ± 0.36 ng/mL and 1.05 ± 0.27 h for cows, respectively. The apparent volume of distribution (V_z_) in pigs and cows was 80.49 ± 43.88 L and 839.88 ± 174.77 L, respectively. The elimination half-time (t_1/2_), and clearance (CL) were 1.29 ± 0.40 h and 41.15 ± 11.18 L/h for pigs and 1.13 ± 0.3 h and 545.04 ± 166.40 L/h for cows, respectively. No adverse effects were observed in any of the animals.

**Conclusion:**

This study extends previous studies describing the pharmacokinetics of buserelin following i.m. administration in pigs and cows. Further studies investigating other factors were needed to establish therapeutic protocol in pigs and cows and to extrapolate these parameters to others economic animals.

## Background

Buserelin, [CAS: 68630–75-1 (C_60_H_86_N_16_O_13_)], is a synthetic analog of gonadotropin-releasing hormone (GnRH) used in the treatment of a variety of hormone disorders. It is more powerful in stimulating the pituitary release of luteinizing hormone (LH) and follicle-stimulating hormone (FSH) than the natural hormone [1, 2]. Therefore, it has been used for the induction of ovulation and improving the conception rates [3–6]. However, when multiple-dose is applied, it produces reversible pituitary desensitization [7]. Therefore, it results in an orchidectomy environment that can be used in the treatment of hormone-sensitive disorders [3]. It has been proved that buserelin is a competitive candidate in the treatment of a variety of hormone-related conditions.

After being approved by the European Medicines Agency (EMA) in 1995 [1], there are some pharmacokinetic studies of buserelin been reported in humans and rats following subcutaneous (s.c.), intranasal (i.n.), or intravenous (i.v.) routes [8–13]. These reported data suggest that high-dose reduces estradiol synthesis and secretion, and inhibits follicular maturation. The EMA has published data following i.v. buserelin and it was found to have a rapid initial half time of 5 min (rats) or 12 min (guinea pigs) [1]. Regardless of the administration route of buserelin, the elimination of half-time is about 72 to 80 min. Protein binding is about 15% [3]. Intact buserelin accumulates in the pituitary gland, liver and kidneys, where its metabolites are degraded and excreted through the urine [14]. Although the clinical use of buserelin in pigs has been previously reported [14], the pharmacokinetics in cows have not been published. Therefore, the plasma pharmacokinetic profile of buserelin in pigs and cows following i.m. administration has become an interesting issue. Two experiments were designed to determine the plasma concentrations of buserelin in pigs and cows, which may provide guidance for the subsequent applications.

## Materials and methods

### Chemicals and materials

Standard buserelin solution (100% purity, 2.00 mg) was purchased from the European Directorate for the Quality of Medicines (EDQM). Acetonitrile (ACN), methanol (Met), and formic acid (FA) were purchased from Fisher Scientific Co. (NJ, USA). All reagents were of the adequate purity (HPLC or higher grade). HPLC water was purified using a Milli-Q synthesis system (Millipore, MA, USA). Other reagents and materials were analytical grade and supplied by the Beijing Chemical Reagent Co. (Beijing, China).

### Standards solutions

A standard stock solution (1 mg/mL) was prepared by dissolving 2 mg standard buserelin in 2 mL methanol. This solution was stored in brown glass bottles at -20 ℃. Working solutions were prepared by diluting the stock solution with methanol.

### UPLC-MS/MS

The UPLC-MS/MS system (Waters Acquity UPLC and Waters Quattro Premier, Waters Co., USA) and the chromatographic column (Agilent Poroshell 120 EC-C_18_, 4.6 × 100 mm, 2.7 µm) were used in this study. The separation was performed with 0.1% formic acid in water (mobile phase solvent A) and 0.1% formic acid in acetonitrile (solvent B) with a flow rate of 0.6 mL/min. The gradient elution program was optimized as follow: 75% A (0–0.5 min), 10% A (5–7 min) 75% A (7.1–10 min). The sample was injected at a volume of 5 µL at 30℃. The mass spectrometer was operated in the positive ion detection mode with the capillary voltage set at 5.5 kV and the source temperature was 550 ℃. Others parameters were as follows: nebulizer gas pressure was 55 psi, ion source gas pressure was 60 psi, curtain gas pressure was 30 psi, declustering potential was 68 V, entrance potential was 10 V, and collision cell exit potential was 13 V.

### Method validation

Selectivity has been assessed by comparing the chromatograms of blank plasma samples and those of corresponding items with buserelin to exclude the interfering peaks. The calibration curve was established by spiking the blank matrix with known concentration of buserelin. Limit of detection (LOD) and Limit of quantification (LOQ) were determined as the concentrations of buserelin which produced signal/ noise ratio of 3 and 10, respectively. For linearity of this method, calibration curves were generated by least squares regression method with a weighting factor and regression coefficient. Recovery was determined by comparing the analytical results of the extracted quality control (QC) samples with pure standard solution. The accuracy was assessed as the percentage of the measured concentration to the nominal standard concentration. The precision was expressed by coefficient of variation. Stability was assessed by autosampler, benchtop, freeze–thaw, and stock solution test. The matrix effect was obtained with the area of post-extraction blank plasma samples added with buserelin at two levels with the equivalent concentration standard solutions that added with initiate mobile phase [15].

### Animal treatments

Five healthy adult female Danish Landrance × Yorkshire × Duroc pigs (100–120 days, 50–60 kg, Ningbo Kuangdai Husbandry Co., Ltd. Ningbo China) and five healthy adult female Holstein cows (1.5–2 years, 440–500 kg, Ningbo Milk Group Co., Ltd. Ningbo China) were randomly selected to use in this research [16]. All animals were examined by a local veterinarian with regard to physical, hematologic, and biochemical conditions during the two-week adaptation period. The two protocols used in this study were reviewed and approved by the Institutional Animal Care and Use Committee of China Agricultural University (pigs: 11605–20-D-007; cows: 11605–20-B-003). Buserelin injection solution (0.5 mg/mL, 10 mL) was acquired from Ningbo Sansheng Pharmaceutical Company. In order to avoid introducing bias results from feeding, each animal in the study fasted for approximately 12 h. Each pig received 1 mg buserelin injection solution (about 0.018 mg/kg), and each cow received 3 mg (about 0.0064 mg/kg). During the entire experiment, water was available ad libitum. Adverse symptoms were recorded and evaluated. Blood samples of 5 mL were collected into heparinized tube (pigs: anterior *vena cava*; cows: *vena jugularis interna*) at 0, 0.083, 0.167, 0.333, 0.5, 0.75, 1, 1.5, 2, 3, 4, 5, 6, 7, and 8 h after i.m. administration. The blood samples were centrifuged for 5 min at 2775 × g. Plasma samples were stored in -20 ℃ until analysis (within 21 days of collection).

### Sample preparation

Frozen plasma samples were thawed and vortexed, and 500 µL plasma and 1 mL 0.1% formic acid in acetonitrile were mixed together. After 10 min ultrasonic treatment, the samples were centrifuged for 10 min at 11,100 × g. The upper layer was collected and the remainder was treated twice. These two supernatants were added together, the samples were evaporated via nitrogen gas at 40 ℃, and redissolved in 2.5 mL 0.1% formic acid in acetonitrile-0.1% formic acid in water (1:3 v/v). After filtered through a 0.22 µm microbore cellulose membrane, the samples were collected and bottled to UPLC-MS/MS system for analysis.

### Data analysis

Plasma concentrations of buserelin were analyzed using the established method, and the pharmacokinetic parameters were calculated via a non-compartmental analysis model 200 in WinNonlin software (WinNolin 8.1.0 Certara, Pharsight, Mountain View, CA, USA) and expressed in mean ± standard deviation (SD), and no statistical tests were applied.

## Results

### Method validation

**Fig. 1:**
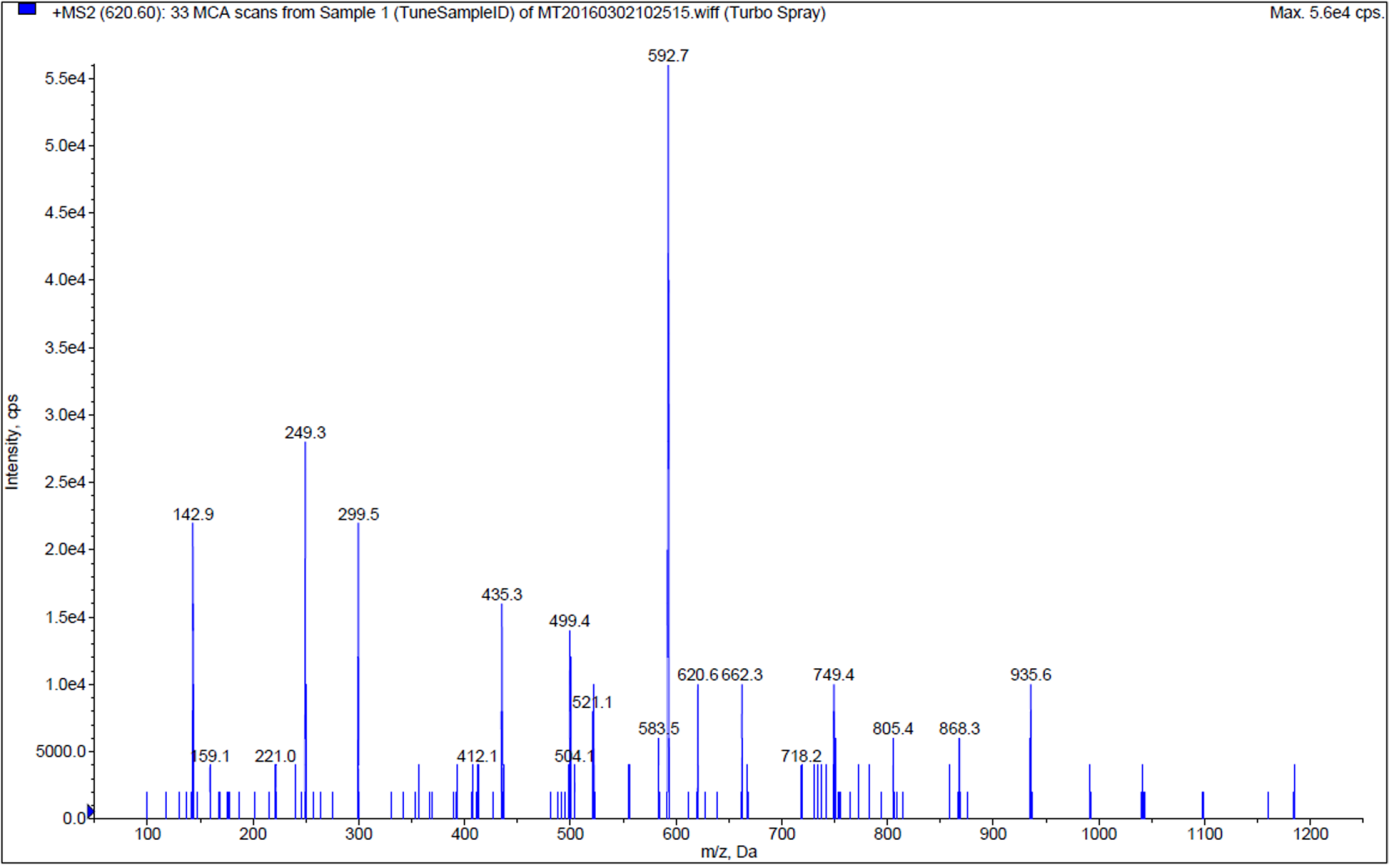
Secondary mass scan of sub ion of buserelin

**Fig. 2:**
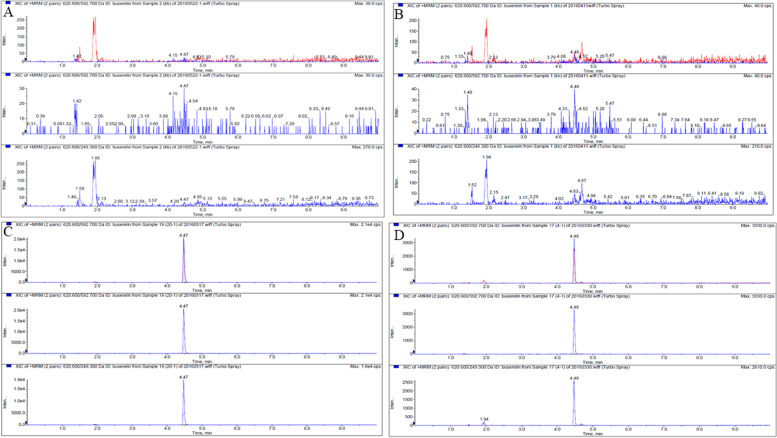
Chromatograms for buserelin. **A** blank pig plasma, **B** blank cow plasma, **C** blank pig plasma spiked with buserelin 200 ng/mL, **D** blank cow plasma spiked with buserelin 40 ng/mL

The quantification and qualitative ions were m/z 620.6 → 592.7 and m/z 620.6 → 249.3, respectively (Fig. 1). With regard to specificity, no interfering signal appeared around the retention time (Fig. 2). The LOD and LOQ were 0.125 ng/mL and 0.25 ng/mL for pigs and 0.0625 ng/mL and 0.125 ng/mL for cows, respectively. The plasma concentration response showed good linearity in the range 0.25–25 ng/mL for pigs, and in the range 0.125–5 ng/mL for cows, respectively. Intra- and inter-assay variabilities were below 15% (Table 1), and the average recoveries ranged from 80 to 120% (Table 2). Buserelin was stable during the assessment of autosampler, benchtop, freeze–thaw, and stock solution test (Table 3). The UPLC-MS/MS method was established and validated according to FDA guidelines on the bioanalytical method validation [15].

**Table 1:** Intra- and inter-assay precision and accuracy of buserelin in pig plasma and cow plasma

Species	Concentration (ng/mL)	Intra-assay precision and accuracy		Inter-assay precision and accuracy	
		Accuracy (%) ± SD	RSD (%)	Accuracy (%) ± SD	RSD (%)
Pig	0.25	92.34 ± 8.06	8.73	96.10 ± 11.63	12.11
	0.5	100.10 ± 5.75	5.74	100.68 ± 8.18	8.13
	10	100.39 ± 4.96	4.94	96.93 ± 5.90	6.09
	20	101.70 ± 6.98	6.87	99.96 ± 7.52	7.52
Cow	0.125	101.00 ± 12.46	12.33	100.44 ± 11.94	11.89
	0.25	102.66 ± 6.73	6.56	101.83 ± 8.55	8.39
	2.5	100.19 ± 7.64	7.62	92.70 ± 7.24	7.82
	4	101.61 ± 7.31	7.20	101.04 ± 6.21	6.15

**Table 2:** Recovery of buserelin from pig plasma and cow plasma

Species	Concentration (ng/mL)	Recovery (%)	
		Mean (%) ± SD	RSD (%)
Pig	0.5	103.68 ± 9.13	8.80
	10	97.38 ± 5.20	5.34
	20	99.99 ± 7.39	7.39
Cow	0.25	106.68 ± 6.91	6.48
	2.5	89.00 ± 3.52	3.96
	4	101.92 ± 5.11	5.02

**Table 3:** Stability of buserelin in pig plasma and cow plasma

Storage conditions	Pig			Cow		
	Concentration (ng/mL)	Accuracy Mean ± SD	RSD (%)	Concentration (ng/mL)	Accuracy Mean ± SD	RSD (%)
Autosampler	0.5	0.51 ± 0.04	8.68	0.25	0.24 ± 0.01	4.07
	20	19.70 ± 2.32	11.79	4	3.90 ± 0.30	7.60
Benchtop	0.5	0.47 ± 0.02	4.91	0.25	0.25 ± 0.02	8.76
	20	19.72 ± 1.61	8.18	4	3.87 ± 0.41	10.54
Freeze–thaw	0.5	0.51 ± 0.04	6.90	0.25	0.26 ± 0.02	6.67
	20	19.95 ± 1.12	5.60	4	3.95 ± 0.34	8.53
Stock solution	100	2.50 ± 0.05 Peak area (10^5^)	1.96	100	2.51 ± 0.05 Peak area (10^5^)	1.97

### Adaptation period

During the two-week adaptation period, local veterinarian had checked conditions of animals using in these two experiments regard to physical, hematologic and biochemical test. The results are as follow in Tables 4, 5, 6 and 7.

**Table 4:** The hematologic results of pigs before pharmacokinetic study

Days	No	Body weight (kg)	RBC	HGB	HCT	MCV	MCH	MCHC	PLT	WBC	LYMPH#
			5.0–8.0 (M/μL)	10.7–16.7 (g/dL)	32–50 (%)	50–68 (fL)	17.0–21.0 (pg)	30.0–34.0 (g/dL)	300–700 (K/ μL)	11.0–22.0 (K/ μL)	6.6–18.7 (K/ μL)
1	1	51.5	5.03	16.68	43.94	65.82	17.57	33.90	642.99	14.77	12.32
	2	53.5	5.33	14.23	45.78	64.19	17.85	33.23	692.16	12.04	8.65
	3	57.0	7.41	15.81	38.01	50.85	18.16	30.23	500.96	12.02	17.63
	4	57.5	5.99	15.34	33.25	51.67	19.44	33.43	495.72	20.14	9.20
	5	55.8	5.06	14.35	41.88	57.80	18.88	32.95	423.13	12.19	12.40
14	1	51.3	6.06	16.63	32.86	55.92	19.01	33.22	533.03	16.92	12.73
	2	57.8	7.66	16.24	49.83	65.28	17.83	33.17	472.42	21.89	14.07
	3	57.0	6.45	14.53	40.77	62.77	19.09	31.83	430.11	14.40	17.27
	4	56.3	5.93	12.09	41.99	59.69	18.95	31.92	557.42	11.70	13.41
	5	57.2	5.51	14.75	37.14	55.01	20.47	30.02	494.97	12.04	15.04

*Abbreviation: RBC* Red Blood Cell, *HGB* Hemoglobin, *HCT* Hematocrit, *MCV* Mean Corpuscular Volume, *MCH* Mean Corpuscular Hemoglobin, *MCHC* Mean Corpuscular Hemoglobin Concentration, *PLT* Platelet, *WBC* White Blood Cell, *LYMPH#* Absolute Lymphocyte

**Table 5:** The hematologic results of cows before pharmacokinetic study

Days	No	Body weight (kg)	RBC	HGB	HCT	MCV	MCH	MCHC	PLT	WBC	LYMPH#
			5.0–10.0 (M/μL)	8.0–15.0 (g/dL)	24–46 (%)	40.0–60.0 (fL)	11.0–17.0 (pg)	30.0–36.0 (g/dL)	230–690 (K/ μL)	4.0–12.0 (K/ μL)	2.5–7.5 (K/ μL)
1	1	455	7.46	11.10	40.12	44.17	11.32	54.08	610.59	9.21	6.79
	2	493	7.79	8.69	29.54	48.70	16.87	55.07	388.85	4.77	2.97
	3	484	6.79	12.49	24.01	44.13	14.41	40.15	598.92	7.93	2.87
	4	442	9.78	8.28	33.38	40.05	15.26	58.64	463.73	8.08	2.59
	5	469	6.56	13.08	42.82	41.55	13.82	37.15	622.68	7.53	2.76
14	1	460	8.57	14.99	29.28	57.21	11.98	56.25	246.81	11.59	6.30
	2	492	5.23	10.60	36.06	47.55	12.26	41.45	382.24	9.99	3.75
	3	481	9.34	11.85	25.18	42.62	13.75	50.60	297.05	4.18	3.88
	4	448	9.82	11.73	30.16	46.07	12.18	58.86	329.32	9.92	3.78
	5	470	7.61	10.20	39.36	55.80	14.72	41.44	417.46	4.78	5.62

*Abbreviation: RBC* Red Blood Cell, *HGB* Hemoglobin, *HCT* Hematocrit, *MCV* Mean Corpuscular Volume, *MCH* Mean Corpuscular Hemoglobin, *MCHC* Mean Corpuscular Hemoglobin Concentration, *PLT* Platelet, *WBC* White Blood Cell, *LYMPH#* Absolute Lymphocyte

**Table 6:** The biochemistry results of pigs before pharmacokinetic study

Days	No	Body weight (kg)	CREA	GGT	ALT	AST	ALP	TP	ALB	TBIL	GLU	Ca	P	TC
			60–110 (μmol/L)	10–60 (U/L)	31–58 (U/L)	32–84 (U/L)	92–290 (U/L)	0–70 (g/L)	0–34 (g/L)	0–11.9 (μmol/L)	3.6–5.4 (mmol/L)	1.63–2.8 (mmol/L)	2.5–3.52 (mmol/L)	0.9–1.40 (mmol/L)
1	1	51.5	84.31	23.18	43.84	1.95	210.87	55.12	23.64	6.52	4.40	2.15	2.98	1.39
	2	53.5	74.34	18.80	47.10	2.70	258.34	55.66	21.47	2.56	5.02	2.18	3.39	1.41
	3	57.0	77.84	20.59	52.00	1.72	133.37	50.05	19.10	2.45	4.00	2.11	2.54	1.24
	4	57.5	65.34	59.74	35.83	1.80	245.52	59.21	19.47	10.35	4.75	1.93	3.26	1.38
	5	55.8	95.88	34.58	54.39	2.74	249.70	62.91	12.10	8.76	3.66	2.29	2.54	1.03
14	1	51.3	72.80	15.99	37.46	0.74	290.22	63.91	21.14	9.23	4.94	1.94	3.23	1.11
	2	57.8	66.53	57.00	43.45	1.03	196.15	68.49	17.53	5.68	5.23	2.82	3.18	1.33
	3	57.0	80.55	30.55	33.72	1.43	207.87	69.98	11.19	3.03	5.08	1.95	3.13	1.07
	4	56.3	101.95	43.46	40.47	1.09	144.23	55.46	5.16	7.10	4.70	2.06	3.00	1.24
	5	57.2	89.60	11.03	45.91	2.58	275.81	66.36	8.27	6.42	4.02	1.78	2.99	1.23

*Abbreviation: CREA* Creatinine, *GGT* Glutamyl-Transpeptidase, *ALT* Alanine Transaminase, *AST* Aspartate Transaminase, *ALP* Alkaline Phosphatase, *TP* Total Protein, *ALB* Albumin, *TBIL* Total Bilirubin, *GLU* Glucose, *Ca* Calcium, *P* Phosphorus, *TC* Total Cholesterol

**Table 7:** The biochemistry results of cows before pharmacokinetic study

Days	No	Body weight (kg)	CREA	GGT	ALT	AST	ALP	TP	ALB	TBIL	GLU	Ca	P	TC
			53–124 (μmol/L)	12–30 (U/L)	11–47 (U/L)	57–108 (U/L)	26–78 (U/L)	0–70 (g/L)	0–34 (g/L)	0–6.8 (μmol/L)	2.8–4.5 (mmol/L)	2–2.5 (mmol/L)	1.5–3.0 (mmol/L)	2.89–8.55 (mmol/L)
1	1	455	118.97	17.20	11.54	65.72	57.90	69.62	23.57	5.58	3.96	2.04	1.94	4.20
	2	493	101.66	29.93	23.10	105.15	32.49	55.18	16.24	4.85	3.82	2.40	2.80	3.78
	3	484	80.64	21.15	25.67	58.87	74.06	56.56	15.15	5.67	2.86	2.49	2.26	8.48
	4	442	64.35	16.29	15.20	99.08	36.72	52.88	16.58	5.85	3.87	2.10	2.76	8.67
	5	469	100.70	28.94	39.54	61.40	44.25	69.12	22.65	4.02	3.47	2.02	2.40	6.57
14	1	460	72.57	19.59	28.39	91.04	66.27	60.60	21.64	5.31	3.89	2.49	2.17	6.70
	2	492	88.87	29.22	40.23	103.90	48.16	66.29	17.09	5.54	2.85	2.46	2.12	3.32
	3	481	65.99	28.76	36.87	73.81	46.36	69.31	18.68	5.46	3.61	2.02	1.56	6.01
	4	448	113.53	18.01	20.58	100.65	64.49	62.20	15.84	5.12	3.99	2.34	1.84	4.57
	5	470	90.39	15.12	16.40	62.28	32.78	67.99	24.84	4.26	4.19	2.36	2.12	3.78

*Abbreviation: CREA* Creatinine, *GGT* Glutamyl-Transpeptidase, *ALT* Alanine Transaminase, *AST* Aspartate Transaminase, *ALP* Alkaline Phosphatase, *TP* Total Protein, *ALB* Albumin, *TBIL* Total Bilirubin, *GLU* Glucose, *Ca* Calcium, *P* Phosphorus, *TC* Total Cholesterol

### Pharmacokinetic study

**Fig. 3:**
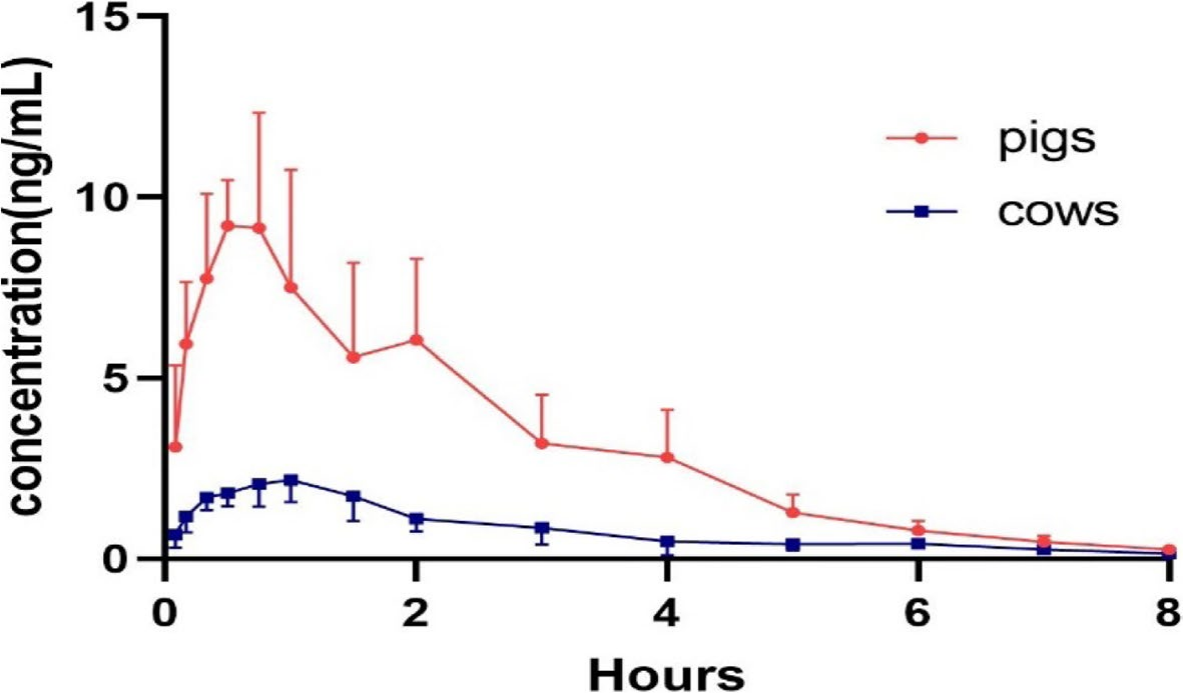
Plasma concentration–time curves of buserelin after i.m. administration of 1 mg per pig and 3 mg per cow

The method described above was successfully applied to quantify buserelin levels in pig plasma and cow plasma. The plasma concentration–time curve of buserelin in pigs and cows is shown in Fig. 3. The major pharmacokinetics parameters of buserelin in pigs and cows are shown in Table 8. For pigs, the AUC_0-t_ was approximately 25.02 ± 6.93 h × ng/mL with C_max_ 10.99 ± 2.04 ng/mL and t_max_ 0.57 ± 0.18 h. V_d_/F was 80.49 ± 43.88 L. The elimination half-time (t_1/2_), and clearance (CL/F) were 1.29 ± 0.40 h and 41.15 ± 11.18 L/h., respectively. For cows, the AUC_0-t_ was 5.63 ± 1.86 h × ng/mL with C_max_ 2.68 ± 0.36 ng/mL and t_max_ 1.05 ± 0.27 h. V_d_/F was 839.88 ± 174.77 L. The elimination half-time (t_1/2_), and clearance (CL/F) were 1.13 ± 0.30 h and 545.04 ± 166.40 L/h., respectively.

**Table 8:** Plasma parameter of buserelin after i.m. administration of 1 mg per pig and 3 mg per cow

Pharmacokinetic parameter	pigs	cows
*t*_max_ (h)	0.57 ± 0.18	1.05 ± 0.27
*C*_max_ (ng/ml)	10.99 ± 2.04	2.68 ± 0.36
*t*_1/2_ (h)	1.29 ± 0.40	1.13 ± 0.30
AUC_0-t_ (h × ng/ml)	25.02 ± 6.93	5.63 ± 1.86
AUC_0-∞_ (h × ng/ml)	25.75 ± 6.75	5.99 ± 2.01
V_d_ /F(L)	80.49 ± 43.88	839.88 ± 174.77
CL/F (L/h)	41.15 ± 11.18	545.04 ± 166.40
MRT_0-t_ (h)	2.13 ± 0.27	1.85 ± 0.56
MRT_0-∞_ (h)	2.35 ± 0.23	2.16 ± 0.70
AUMC_last_(h × h × ng/mL)	59.59 ± 16.54	11.23 ± 6.89
C_max_/D(ng/mL/mg/kg)	610.19 ± 79.93	420.35 ± 58.40
k_a_(h^−1^)	0.48 ± 0.07	0.58 ± 0.17

*Abbreviation: t*_*max*_ time to peak concentration, *C*_*max*_ maximum plasma concentration, *t*_*1/2*_ elimination half-time, *AUC*_*0-t*_ Area Under The Concentration–time curve from 0 to the last measurement point, *AUC*_*0-∞*_ Area Under The Concentration–time curve from 0 to infinity, *V*_*d*_*/F* apparent Volume of distribution of Fraction absorbed, *CL/F* plasma Clearance of Fraction absorbed, *MRT*_*0-t*_ Mean Residence Time from 0 to the last collection point, *MRT*_*0-∞*_ Mean Residence Time from 0 to infinity, *AUMC* the total Area Under The Moment Curve from the time of dosing to the last measurable concentration, *C*_*max*_*/D* the rate of Cmax to Dosage, *k*_*a*_ absorption rate constant

## Discussion

Previous studies using immunohisoflurescence or radioimmunoassay had demonstrated to evaluate the concentration of buserelin in plasma However, antibody preparation is included in this method, which is very time consuming [7, 17, 18]. Reverse phase high-performance liquid chromatography (RP-HPLC) was used for analysis of gonadorelin analogues. But an ion pair agent was need which trifluoracetic acid created a low pH environment [19, 20]. Capillary electrophoresis (CE), a powerful tool, was used to analyze the peptide. However, a high ionic strength of buffer was selected to decrease absorption of buserelin in the analysis [21–23]. Several technical tools were combined with CE to detect the concentration. These methods can speed up the quantification of buserelin, but pH 3.0 was needed for successful separation of the solution [23, 24]. The HPLC method reduces the retention time from 40 min to 9.2 min [25]. Currently, UPLC-MS/MS is used to detect peptides and proteins for doping control. The sample preparation was dilute-shoot (DS) or solid-phase extraction (SPE), which will be cost-effectiveness and loss of sensitivity [26]. In our research, a rapid and sensitive UPLC-MS–MS method was established and validated following FDA guidance to evaluate busesrelin levels in blood samples.

In this study, t_max_ was achieved after 0.57 ± 0.18 h for pigs and 1.05 ± 0.27 h for cows which is longer than reported buserelin solution in rats (45 min) at a single dose of 6 mg/kg following s.c. administration [13], health volunteers (20 min, 42 min, 58 min, 43.8 min, 38.8 min) after being administered at a single dose of 500 µg i.v., 5 µg s.c., 150 µg i.n., 300 µg i.n., and 450 µg i.n., respectively [3], and shorter than buserelin suspension in rats (180 min) at 6 mg/kg dose following s.c. administration, rats (1.92 ± 0.42 h) at a single dose of 0.1 mg/kg i.n., and dogs (4 h) after being administered s.c. at a dose of 3.3 mg [18]. The t_max_ reflects the rate of absorption, which indicated that buserelin in pigs was absorbed very quickly due to the rate of metabolic rate of organs to the whole body [27]. These differences in the parameters show that buserelin is absorbed at a faster rate in pigs than in cows. These data agree with the view that small animals eliminate the drugs more rapidly than large ones. However, compared the data of health volunteers with the one of rats following i.n., the conclusion seems paradox. It can be explained by the fact that rats using in the experiment were anesthetized. The physical condition can affect absorption progress.

The V_d_ was 80.49 ± 43.88 L for pigs and 839.88 ± 174.77 L for cows, which is larger than pigs (304 ± 112 mL/kg) at a single dose 1 mg i.v. [14], and dogs (50.1 ± 2.4 mL/kg) at a single dose 5 mg i.v. [18]. High plasma concentration, high bonding rate, means more drugs cannot across the membrane and barrier. Therefore, binding changes can affect the distribution of drugs. Because protein binding of buserelin is about 15% [2], it is proportional to the body volume and body weight and animals using in this study had larger volume of body water or extracellular water [27]. It has been reported that buserelin is rapidly degraded by pyroglutamyl-amino-peptidase which can be isolated from mammalian liver [2]. The main serum metabolite was buserelin (5–9) pentapeptide [3]. Its intact form and metabolites are mainly excreted through urine [14, 27]. This view has been proved correct when compared with the clearance of buserelin solution in rats (30.34 ± 2.12 mL/min) at 6 mg/kg s.c. injection [13], in dogs (1.7 ± 0.10 mL/kg/min) at 5 mg per dog i.v. injection [18], and in pigs (2.0 ± 0.4 mL/kg/min) at 1 mg per pig i.v. injection These data show that hepatic blood flow is the major determinants for the elimination process because of it has an allometric relationship with body weight [27]. The elimination half-time is proportional to its volume of distribution, but inversely to its clearance. The t_1/2_ value was in pigs 1.29 ± 0.40 h and in cows 1.13 ± 0.30 h which is longer than in rats (5 min) in guinea pigs (12 min) following i.v. application [1], in rats (42 min) at a single dose of 6 mg/kg s.c. injection [13] and in dogs (56.4 ± 0.98 min) at a single dose of 5 mg i.v. injection [18], approximately equate to the value in pigs (103 ± 20 min) at a single dose of 1 mg i.v.injection [14]. However, the elimination half-time of buserelin in human has a 72–120 min regardless of the administration route [2, 3]. These data show that pigs and cows have a low elimination process which can be related with the rate of metabolism.

Similar to other peptide hormones, buserelin after oral administration will be largely digested. The pharmacokinetic data obtained following oral administration showed a short half-life and a rapid clearance due to degradation into smaller metabolites without biological activity. However, there are some attempts which can slow-down absorption from delivery site or slow-down enzymatic degradation and elimination to improve the bioavailability, such as sodium glycodeoxycholate, Zn^2+^ suspension within the buserelin solution, and cyclodextrin derivatives [13, 14, 17]. Meanwhile some reports hold the view that buserelin administration induces loss of erectile potency, hot flush [9], uterine bleeding [6], apoptosis in spermatozoa lineage and inhibits immune system function [28, 29], further investigations are required to assess its side effects in practical applications.

## Conclusion

In this paper, a sensitive and rapid stability UPLC-MS/MS method has been established and was applied to evaluate the pharmacokinetics of buserelin in pigs and cows after i.m. administration. This is the first to investigate the pharmacokinetic parameters of buserelin in cows and will provide a basis for further study.

## Data Availability

All data generated or analyzed during this study are availability from the corresponding author on reasonable request.

## Abbreviations

LHRH: Luteinizing hormone releasing hormone
UPLC-MS/MS: Ultra-performance liquid chromatography tandem mass spectrometry
i.m.: Intramuscular
s.c.: Subcutaneous
i.n.: Intranasal
i.v.: Intravenous
RBC: Red blood cell
HGB: Hemoglobin
HCT: Hematocrit
MCV: Mean corpuscular volume
MCH: Mean corpuscular hemoglobin
MCHC: Mean corpuscular hemoglobin concentration
PLT: Platelet
WBC: White blood cell
LYMPH#: Absolute lymphocyte
CREA: Creatinine
GGT: Glutamyl-transpeptidase
ALT: Alanine transaminase
AST: Aspartate transaminase
ALP: Alkaline phosphatase
TP: Total protein
ALB: Albumin
TBIL: Total bilirubin
GLU: Glucose
Ca: Calcium
P: Phosphorus
TC: Total cholesterol
t_max_: Time to peak concentration
C_max_: Maximum plasma concentration
t_1/2_: Elimination half-time
AUC_0-t_: Area under the concentration–time curve from 0 to the last measurement point
AUC_0-∞_: Area under the concentration–time curve from 0 to infinity
V_d_/F: Apparent volume of distribution of fraction absorbed
CL/F: Plasma clearance of fraction absorbed
MRT_0-t_: Mean residence time from 0 to the last collection point
MRT_0-∞_: Mean residence time from 0 to infinity
LOD: Limit of detection
LOQ: Limit of quantification
HPLC: High performance liquid chromatography
QC: Quality control
CE: Capillary electrophoresis
